# Analysis of Chemical Composition and Odor Characteristics in Particleboards Decorated by Resin-Impregnated Paper, Polypropylene Film and Polyvinyl Chloride Film

**DOI:** 10.3390/polym17152145

**Published:** 2025-08-05

**Authors:** Liming Zhu, Minghui Yang, Lina Tang, Qian Chen, Xiaorui Liu, Xianwu Zou, Yuejin Fu, Bo Liu

**Affiliations:** Research Institute of Wood Industry, Chinese Academy of Forestry, National Center for Quality Supervision and Testing of Wood and Bamboo Products, Beijing 100091, China; izhulm@163.com (L.Z.); ymh13941135183@163.com (M.Y.); 18211090798@163.com (L.T.); chenqian0610@126.com (Q.C.); 18852003990@163.com (X.L.); xwzou@caf.ac.cn (X.Z.); bj-fyj@163.com (Y.F.)

**Keywords:** resin-impregnated paper, PVC film, PP film, particleboard, VOCs, odorant

## Abstract

Analysis of changes in TVOC and VOCs chemical composition or odor characteristics of particleboard before and after decoration treatment with resin-impregnated paper (RIP), polypropylene (PP) film and polyvinyl chloride (PVC) film were studied. The effects of these three decoration treatments on masking or suppressing the release of VOCs and odorants from particleboard were explored. The substances that were covered or suppressed and newly introduced before and after processing were identified to provide a basis for reducing the odor emissions of PVC-, PP- and RIP-decorated particleboard. Taking undecorated particleboard and particleboard treated by three types of decorative materials as research subjects, the air permeability of the three decorative materials was tested using the Gurley Permeability Tester. TVOC emissions from the boards were evaluated using the 1 m^3^ environmental chamber method. Qualitative and quantitative analyses of the samples were conducted via thermal desorption–gas chromatography–mass spectrometry (TD-GCMS). The contribution of odor substances was determined using odor activity value (OAV). The results indicated that the permeability from high to low was PVC film, PP film and RIP. Compared with undecorated particleboard, the TVOC emissions of RIP-decorated boards decreased by 93%, PP-decorated particleboard by 83% but the TVOC emissions of PVC-decorated particleboard increased by 67%. PP decoration treatment masked or suppressed the release of 20 odor substances but introduced xylene, which can increase potentially the health risks for PP-decorated particleboard. PVC decoration treatment masked or suppressed 19 odor substances, but it introduced 12 new compounds, resulting in an overall increase in TVOC emissions. RIP treatment did not introduce new odor substances. After PP film and RIP treatments, both the variety of VOCs released and the number of key odor-contributing compounds and modifying odorants decreased. In contrast, the number of modifying odorants and potential odorants increased after PVC treatment. VOC emissions were effectively masked or suppressed by three decoration treatments, same as the release of substances contributing to overall odor of particleboard was reduced. Among them, PP and RIP decorative materials demonstrate better effects.

## 1. Introduction

According to statistics, 80% of a person’s life is spent indoors, so people are increasingly concerned about the safety and protection of indoor environments [1]. United Nations data indicate that globally, over 300 million people are homeless and more than 2.8 billion live in substandard housing conditions. Achieving a green living environment is pivotal for advancing sustainable development goals, necessitating the development of novel building materials with low odor and minimal volatile organic compound (VOC) emissions [2]. Particleboard is a widely used indoor decoration material, valued for its lightweight, moderate strength and diversified size specifications, which has been unprecedentedly applied in customized furniture [3]. However, with the increasing utilization of particleboard, issues such as high concentrations of volatile organic compounds emitted from indoor building materials and associated odor problems have raised concerns among consumers. These issues impact human health, potentially inducing Sick Building Syndrome (SBS) symptoms such as headaches, drowsiness, nausea and runny nose [4]. Engineered wood products are often surface-decorated in decoration applications. For particleboard, surface decorating primarily involves overlaying decorative materials. Common decorative materials for wood-based panels include resin-impregnated paper (RIP), polyvinyl chloride (PVC) film and polypropylene (PP) film [5,6]. Decorated particleboard offers advantages such as cost-effectiveness, uniform structure, wear-resistant surface, high-temperature tolerance and corrosion resistance, and is widely used in indoor decoration and production of panel furniture.

Existing studies have explored pollutant emissions from decorative materials and decorated boards. For instance, Li and Wang analyzed odor emissions and environmental impacts of melamine-impregnated paper-decorated medium-density fiberboard and lacquered particleboard [7,8,9]. Liu et al. investigated the influence of various decorative materials (e.g., PP film, UV-cured coatings, melamine-impregnated paper) on formaldehyde emissions [7]. While most current research focuses on VOC composition changes before and after surface decorating, studies on the impact of VOC release primarily concentrate on either single materials or singular metrics (e.g., TVOC emission levels). After decorating treatments, certain VOCs or odorants may be masked, while the decorating materials themselves may introduce new VOCs or odorants [10]. Moreover, existing research shows insufficient attention to odor issues.

Therefore, this study investigates three types of particleboards treated with distinct decorating materials. By integrating air permeability testing, TVOC emission quantification and qualitative/quantitative analysis via thermal desorption–gas chromatography–mass spectrometry (TD-GCMS), we systematically examine the comprehensive effects of decorating materials on the release of VOCs and odorants—including masked/suppressed substances, newly introduced compounds, and their contributions to odor profiles. Through odor activity value (OAV) and risk index analyses, this work quantitatively assesses changes in key odor contributors, modifying odor components and material risk indices following different decorating treatments. This provides a scientific basis for odor risk assessment in decorating material selection. Furthermore, by correlating air permeability measurements with VOC emission data, we reveal the relationship between the gas permeability of decorating materials and their impact on VOC release, offering theoretical support for optimizing decorating material performance.

## 2. Materials and Methods

### 2.1. Materials

The experimental materials, including PP film, PVC film, melamine-impregnated overlay paper, PVC-decorated particleboard, PP-decorated particleboard, RIP-decorated particleboard and their undecorated particleboards, were all provided by a custom furniture enterprise in Guangzhou.

**Figure 1:**
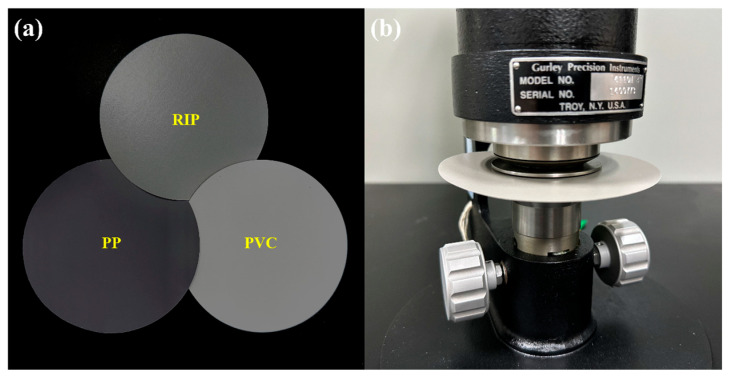
Three types of decorative materials and the air permeability meter. (**a**) RIP, PP and PVC; (**b**) air permeability measurement experiment.

According to experimental requirements, circular specimens with a diameter of 50 mm were taken from the three types of decorative materials. The Gurley method was employed to measure the air permeability of the three materials. Clamp the sample between two cylinders and use the gravity of the cylinders to compress the air inside the cylinders, causing the cylinders to descend after the air passes through the sample, as shown in Figure 1. By measuring the time it takes for 100 mL of air to pass through the tested piece, the air permeability of the three materials can be calculated. 

The sample particleboards were cut into dimensions of 500 mm (length) × 500 mm (width) × 18 mm (thickness). According to HJ 571-2010 [11] “Technical requirement for environmental labeling products—wood based panels and finishing products” (Standard of the Chinese Ministry of Ecology and Environment), all particleboard specimens were prepared for double-side emission testing, with all four edges sealed using aluminum foil tape.

### 2.2. Equipment

The air permeability meter is manufactured by Beijing Guanghua Qimingfeng Technology Co., Ltd. in Beijing, China; the thermal desorption–gas chromatography–mass spectrometry is manufactured by Shimadzu Corporation in Kyoto, Japan.

### 2.3. Methods

#### 2.3.1. Air Permeability of Decorative Materials

The air permeability of three kinds of decorative materials was tested with the Gurley air Permeability Tester. Each sample was tested 5 times on the front and back sides and the average value of 10 measurement results was taken as the final air permeability data.

#### 2.3.2. VOCs Emission of Undecorated Particleboard and Surface-Finished Particleboards

The samples were placed into a 1 m^3^ VOC emission chamber, with the temperature adjusted to 23 ± 0.5 °C and relative humidity 45 ± 3%. After circulating for 72 ± 2 h, the air inside the chamber was sampled using TENAX adsorption tubes at a flow rate of 500 mL/min for a duration of 12 min.

The VOCs emitted from materials in the VOC chamber were analyzed by Shimadzu thermal desorption–gas chromatography–mass spectrometry (TD-GCMS). The thermal desorption conditions were as follows: cold trap adsorption temperature at −10 °C, helium carrier gas flow rate 60 mL/min, desorption temperature 280 °C, transfer line temperature 250 °C, desorption duration 5 min, pre-purge time 1 min and injection time 1 min. For gas chromatography, a DM-TVOC quartz capillary column (50 m × 0.32 mm × 1 μm) was used. The injector temperature was set at 250 °C with helium carrier gas at a flow rate of 1.53 mL/min. The temperature program comprised an initial temperature of 50 °C held for 2 min, ramped at 10 °C/min to 120 °C and held for 10 min; then increased at 5 °C/min to 160 °C and held for 10 min; further ramped at 5 °C/min to 200 °C and held for 10 min; finally raised at 5 °C/min to 250 °C and held for 5 min. Mass spectrometry conditions included interface temperature 280 °C, quadrupole temperature 150 °C, EI ion source temperature 230 °C, electron energy 70 eV and mass scan range 45–550 m/z. Qualitative analysis of VOCs and odor-active compounds was performed using the NIST database (Version 17.0). Quantitative analysis was conducted via external standard calibration with toluene as the reference substance.

#### 2.3.3. Odor Activity Value

The odor activity value (OAV) is defined as the ratio of a compound’s concentration to its odor threshold, serving as an effective indicator for evaluating the contribution of odor compounds to overall odor characteristics. It is generally believed that when the odor substance OAV > 1, the substance is defined as the key odor component, which has a direct impact on the overall gas; When 0.1 < OAV < 1, it is defined as a modified odor component, which can modify the overall odor; when OAV < 0.1, it is defined as potential odor component, which has no significant effect on flavor. The greater the OAV within a certain range, the greater the contribution of this component to the overall odor. By consulting relevant books, chemical book websites, the NIST database and the relevant literature, the odor thresholds and odor types of various substances in VOCs of undecorated and decorated particleboards were found and determined. Calculate the OAV of the substance according to Formula (1).

(1) $$OAV=\frac{C}{OT}$$

where:

OAV—odor activity value (dimensionless);
C—mass concentration of the volatile organic compound, mg/m^3^;
OT—odor threshold of the volatile organic compound, mg/m^3^.

In addition to calculating the odor activity value (OAV), many researchers quantitatively assess the risk of odor-active compounds in VOCs using the lowest concentration of interest (LCI) as an objective basis for hazard evaluation [12]. The German Committee for Health-related Evaluation of Building Products (Ausschuss zur gesundheitlichen Bewertung von Bauprodukten, AgBB) and the EU Report No. 29 have systematically compiled LCI values for over 200 substances.

Based on LCI values retrieved from the literature, the risk value (*R_i_*) of a VOC is calculated using Formula (2):

(2) $$R_{i}=\frac{C_{i}}{{LCI}_{i}}$$

where:

*R_i_* = risk value (dimensionless);
*C_i_* = mass concentration of a specific volatile organic compound in the panel, mg/m^3^;
*LCI_i_* = LCI value of the corresponding volatile organic compound, mg/m^3^.

## 3. Results and Discussion

### 3.1. Permeability of Decorative Materials

Air permeability refers to a material’s ability to allow air to pass through, with higher permeability indicating greater pore density within the material. For decorative materials, this property not only influences physical characteristics such as moisture absorption and water resistance but also significantly impacts adhesion performance to substrates [13]. Consequently, precise control of air permeability is essential in both the production and application of decorative surface materials.

**Figure 2:**
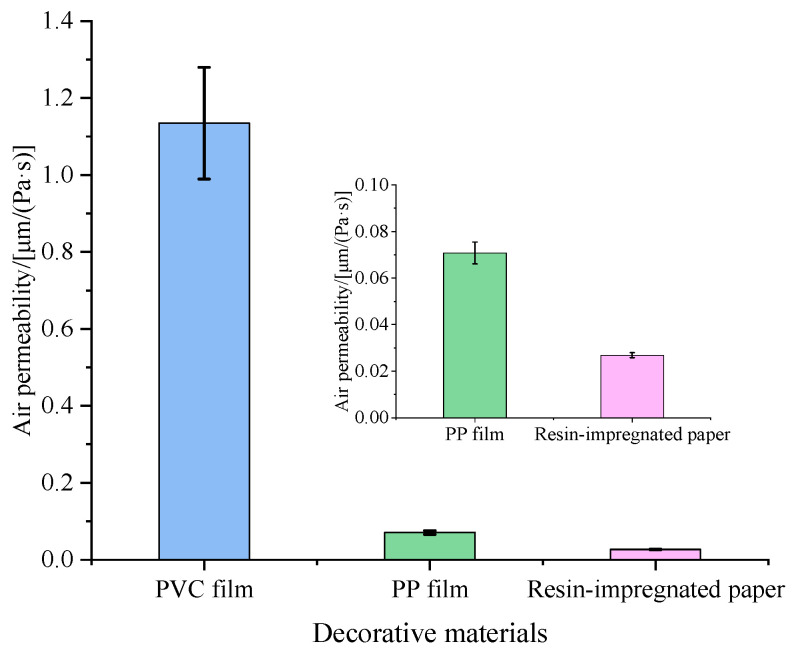
Air permeability of three decorative materials.

The release process of pollutants from wood-based panels can generally be divided into three stages: internal diffusion within the panel, gas-solid interface partitioning and dispersion into the air layer [14]. The addition of decorative materials and their permeability characteristics significantly influence the distribution coefficient of pollutants in the gas-solid interface layer, thereby affecting the overall release dynamics. Higher material permeability corresponds to greater surface porosity and a lower pollutant partition coefficient, promoting easier pollutant emission [15,16,17]. Figure 2 illustrated the permeability test results of three decorative materials. 

PVC film exhibits significantly higher air permeability compared to PP film and RIP, which may be attributed to its inherent pore structure and chemical properties. The higher crystallinity and non-polar characteristics of PVC typically result in increased surface porosity, contributing to its enhanced permeability [13,18]. Conversely, RIP undergoes impregnation and coating processes during production, where resin filling substantially reduces pore density and drastically diminishes air permeability [19,20]. 

### 3.2. Emission Analysis of VOCs Released from Undecorated Particleboard and Three Decorated Particleboards

**Figure 3:**
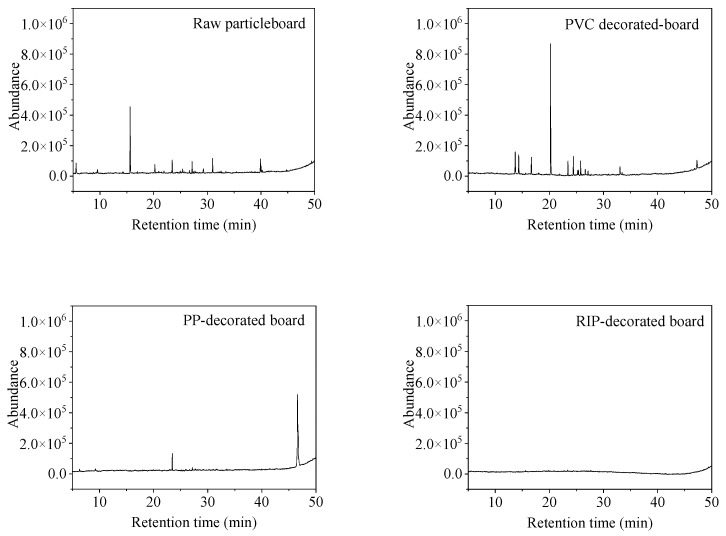
Total ion current (TIC) of VOCs released from undecorated and decorated particleboards.

**Figure 4:**
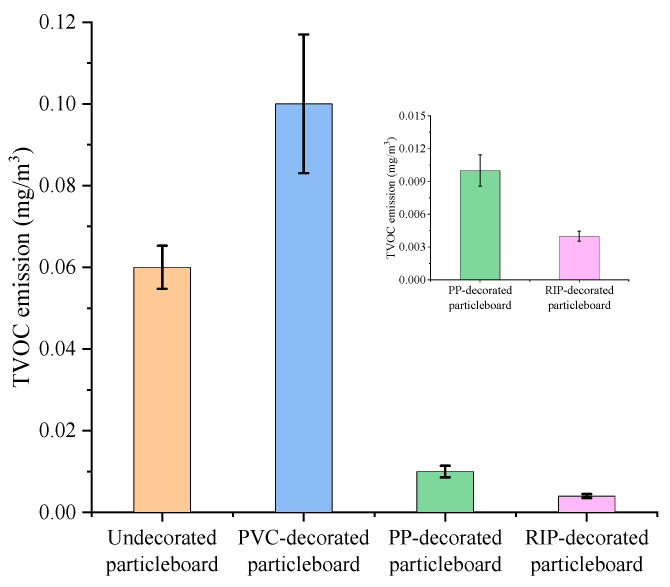
Changes in TVOC emission of undecorated and decorated particleboards.

The total ion current (TIC) chromatograms of undecorated particleboard, PP-decorated particleboard, PVC-decorated particleboard and RIP-decorated particleboard were shown in Figure 3. The TVOC emission levels before and after decoration treatments were illustrated in Figure 4 and detailed chemical compositions and emission concentrations of VOCs are listed in Table 1.

Testing standards for wood-based panels commonly adopted in China, the EU, the United States, Japan and other regions are summarized in Table 2. For the EU, US and Japan, key standards include ISO 16000-9 [21], ISO 16000-6 [22], EN 16516 [23], ANSI/BIFMA M7.1 [24], and JIS A1901 [25]. Among these, only JIS A1901 specifies a TVOC emission limit of ≤0.4 mg/(m^2^·h) using the small chamber method; all others are methodology standards focusing exclusively on testing procedures (e.g., sampling, chamber conditions) with no emission limits defined, requiring integration with regional regulations for compliance. In China, primary standards encompass HJ 571-2010 [11], GB/T 29899-2024 [26], GB/T 35601-2024 [27] and GB/T 44690-2024 [28]. GB/T 29899-2024 [26], equivalent to ISO 16000 [21,22] principles, details small chamber method parameters (temperature, humidity, air exchange rate) but sets no emission limits, serving solely as a testing protocol. Conversely, HJ 571-2010 [11], GB/T 35601-2024 [27] and GB/T 44690-2024 [28] establish TVOC emission thresholds for various products.

After 72 h of emission testing in a 1 m^3^ VOC chamber, the TVOC release concentrations of undecorated particleboard, PVC-decorated particleboard, PP-decorated particleboard and RIP-decorated particleboard were 0.06 mg/m^3^, 0.10 mg/m^3^, 0.01 mg/m^3^ and 0.004 mg/m^3^, respectively. The TVOC emission levels of each type of panel are consistently low, with all meeting or even exceeding the Green Benchmark tier specified in GB/T 35601-2024 [27].

Compared with the undecorated particleboard, the TVOC release of PP-decorated and impregnated paper decorated particleboard decreased by 83% and 93%. Decoration treatment partially inhibits VOC emissions from the base particleboard, primarily because the lower surface porosity of decorative materials effectively seals internal VOC release pathways [29].

While after PVC decoration treatment, the TVOC emission increased by 67%. This paradoxical rise can be attributed to two factors. Firstly, compared to the other two materials, PVC has higher air permeability, allowing pollutants to escape more easily from the internal structure of the particleboard. Secondly, PVC itself releases pollutants, which may introduce additional contaminants (e.g., plasticizers or stabilizers) during the laminating process, resulting in an increase in the final TVOC release instead of a decrease.

As shown in Table 1, most detected VOCs, such as n-pentanal, n-hexanal, eucalyptol, linalool, α-terpineol, cedrol, longifolene and α-pinene, originated from the wood itself. Ketones and alcohols likely derive from the degradation of wood fibers and lipids [30,31,32], while aldehydes are generated by the breakdown of cellulose, hemicellulose and low-molecular-weight carbon-containing compounds [33]. Additionally, adhesives and auxiliary agents (e.g., defoamers, dispersants and fragrances) used during production contribute aromatic hydrocarbons, aldehydes, alcohols and ketones [34]. Overall, the VOCs emitted from both uncoated and surface-treated particleboards exhibit diverse and complex sources.

From Table 1, it can be observed that the shared VOCs released from undecorated particleboard and PP-decorated boards include nonanal, 2-ethylhexanol, eucalyptol, acetophenone and α-pinene. After decoration, the emission levels of these shared substances decreased except for α-pinene. Compared to undecorated particleboard, PP decoration shielded or suppressed the release of 20 substances but introduced xylene, which may increase the health risks associated with PP-decorated particleboards. For the shared VOCs between undecorated particleboard and PVC-decorated boards, the emission quantity of eucalyptol and acetophenone decreased after decoration, while α-pinene emission slightly increased and toluene emission increased by more than 20 times. PVC decoration covered or suppressed 19 substances but introduced 12 new substances, including decanal, n-hexanal, 2-ethylhexanol, 2-propyl-1-heptanol, cyclohexanone, hydroxyethyl acrylate, butyl acetate, 2-ethylhexyl acetate, isooctyl acrylate, sec-butyl acetate, o-xylene and phenol. Consequently, the TVOC emission of PVC-decorated particleboards exceeded those of undecorated particleboards. In contrast, impregnated film paper decoration reduced the emission of shared substances such as benzaldehyde and acetophenone, covered or suppressed 19 substances and no new substances were introduced.

**Figure 5:**
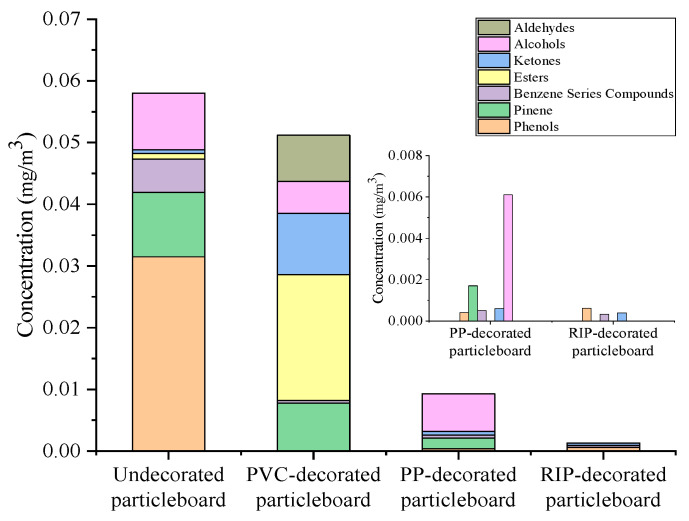
Changes in the release of various VOCs of particleboard.

The concentration changes of various types of VOCs before and after particleboard decoration treatment were illustrated in Figure 5 and Table 3. Aldehydes account for a higher proportion of VOCs released from undecorated particleboard, esters from PVC-decorated particleboard and terpenes from PP-decorated particleboard. Previous studies primarily focused on proportional changes in VOC categories (e.g., esters, alkenes, alcohols) before and after decoration, which failed to reflect actual emission quantity variations.

As shown in Table 3, compared to undecorated particleboard, impregnated film paper decoration effectively suppressed all VOC categories except benzene series compounds. Among the VOCs by PP-decorated particleboard, except for pinene, which decreased by 34% and benzene series compounds, the emissions of other substances decreased by more than 80%. In contrast, PVC-decorated particleboard demonstrated a 25% decline in alcohols, a 43% reduction in pinenes and an over 90% decrease in aldehydes and ketones. However, benzene series compounds and esters increased by approximately 16-fold and 22-fold, respectively. Phenolic compounds were introduced, which were absent in undecorated particleboards. These factors contributed to a 67% increase in TVOC emissions for PVC-decorated particleboard.

Additionally, as shown in Figure 2, PVC film exhibited significantly higher gas permeability than PP film and impregnated film paper, with larger surface pores and lower resistance to VOC release from the substrate. This structural characteristic explained why PVC-decorated particleboard yielded higher TVOC emissions than the other two decorated types.

From the above analysis, it can be clearly observed that in terms of suppressing VOCs release, the impregnated film-decorated paper demonstrated the best performance, followed by PP film, while PVC film showed the worst effectiveness.

### 3.3. OAV of VOCs in Particleboard and Three Decorated Particleboards

The OAV of VOCs in undecorated, PVC-decorated, PP-decorated and RIP-decorated particleboards are presented in Table 4. In total, 21 VOCs were detected in undecorated particleboard. The OAVs of 14 substances can be calculated based on literature-reported olfactory thresholds. Four compounds exhibited OAV > 1, including n-hexanal (sharp grassy/apple fragrance, OAV = 24.2727), n-octanal (fruity aroma, OAV = 22.0000), longifolene (woody/iris-like aroma, OAV = 1.8333) and n-pentanal (herbaceous note, OAV = 1.4286). Four substances fell within the 0.1 < OAV < 1 range, while six showed OAV < 0.1. In total, 19 VOCs were detected in PVC-decorated particleboard. The OAVs of 14 substances were calculated, which only sec-butyl acetate (pleasant aroma, OAV = 1.0351) exhibited OAV > 1. Five substances were in the 0.1 < OAV < 1 range and eight showed OAV < 0.1. Seven VOCs were detected in PP-decorated particleboard. The OAVs of six substances were determined, with three each in the 0.1 < OAV < 1 and OAV < 0.1 ranges. RIP -decorated particleboard showed seven detected VOCs. The OAVs of five substances were calculated. After surface treatment, only n-hexanal (sharp grassy/apple fragrance, OAV = 4.3174) exhibited OAV > 1, while two substances each occupied the 0.1 < OAV < 1 and OAV < 0.1 ranges.

### 3.4. Risk Values of Odorants in Particleboard and Three Decorated Particleboards

The risk values of various VOC substances calculated according to Formula (2) are listed in Table 5, showing that in undecorated particleboards, substances with higher risk values include n-hexanal (0.0297), n-pentanal (0.0025) and 2-ethylhexanol (0.0023); in PVC-decorated particleboards, substances with higher risk values include phenol (0.7500), cyclohexanone (0.1227) and toluene (0.0032); in PP-decorated particleboards, substances with higher risk values include α-pinene (0.7500), 2-ethylhexanol (0.0020) and xylene (0.0012); in RIP-decorated particleboards, substances with higher risk values include n-hexanal (0.0052), acetophenone (0.0010) and xylene (0.0008). Overall, PVC-decorated particleboards exhibit higher risk values, primarily due to the introduction of the new pollutant phenol. Although the pollutants released by these panels include moderately and low-toxicity substances, their risk values are significantly less than 1, indicating no significant hazard to human health.

## 4. Conclusions

(1) PP and RIP exhibit significantly lower air permeability than PVC, providing superior blocking effects that effectively reduce TVOC emissions from particleboards, while also diminishing key odorants and odor-modifying compounds.
(2) Surface decorating not only blocks or suppresses VOC emissions from particleboards but simultaneously introduces new VOCs from the decorating materials themselves, potentially increasing the panels’ usage risks.
(3) PVC-decorated particleboards demonstrate higher risk values, primarily due to the introduction of phenol as a new pollutant. Although the emitted contaminants from these panels include substances of low-to-moderate toxicity, their risk values remain well below 1, posing no significant health risks to humans.
(4) In subsequent research, finishing materials and wood-based panels from different countries or regions should be selected for testing to enhance the regional applicability of the conclusions. Additionally, it is essential to continue supplementing data on the odor thresholds and LCI values of VOCs and establish a comprehensive database.

## Figures and Tables

**Table 1:** Chemical composition and release of VOCs before and after decoration.

Substance	CAS Number	Compound	Concentration			
			Undecorated Particleboard	PVC-Decorated Particleboard	PP-Decorated Particleboard	RIP-Decorated Particleboard
Aldehydes	110-62-3	n-pentanal	0.0020	—	—	—
	66-25-1	hexanal	0.0267	0.0004	—	0.0047
	111-71-7	heptaldehyde	0.0004	—	—	—
	124-19-6	nonanal	0.0010	—	0.0004	0.0006
	2548-87-0	trans-2-octenal	0.0003	—	—	—
	124-13-0	n-octanal	0.0011	—	—	—
	112-31-2	decanal	—	0.0003	—	—
	100-52-7	benzaldehyde	—	—	—	—
Alcohols	71-41-0	1-pentanol	0.0004	—	—	—
	96-23-1	1,3-dichloropropanol	0.0032	—	—	—
	104-76-7	2-ethylhexanol	0.0007	0.0022	0.0006	—
	470-82-6	eucalyptol oil	0.0040	0.0015	0.0011	—
	78-70-6	linalool	0.0012	—	—	—
	98-55-5	α-terpineol	0.0005	—	—	—
	8000-27-9	cedrol	0.0004	—	—	—
	10042-59-8	2-propyl-1-heptanol		0.0038	—	—
	20324-32-7	1- (2-methoxy-1-methylethoxy) isopropanol		—	—	0.0007
Ketones	98-86-2	acetophenone	0.0006	0.0004	0.0005	0.0005
	76-22-2	2-camphanone	0.0048	—	—	—
	108-94-1	cyclohexanone	—	0.0503	—	—
	513-86-0	3-hydroxy-2-butanone	—	—	—	0.0059
Esters	123-86-4	butyl acetate	—	0.0065	—	—
	31565-19-2	2-ethylhexyl acetate	—	0.0004	—	—
	29590-42-9	isooctyl acrylate	—	0.0010	—	—
	105-46-4	sec-butyl acetate	—	0.0118	—	—
	818-61-1	hydroxyethyl acrylate	0.0006	0.0007	—	—
	54774-91-3	acrylic acid, 6-methylheptyl ester	0.0003	—	0.0005	—
Benzene Series Compounds	108-88-3	toluene	0.0004	0.0094	—	—
	100-41-4	ethyl benzene	—	0.0004	—	—
	95-47-6	o-Xylene	—	0.0005	0.0006	0.0004
	108-38-3	m-Xylene				
	106-42-3	p-Xylene				
	99-87-6	4-isopropyl toluene	0.0002	0.0003	—	—
Pinene	475-20-7	longifolene	0.0044	—	—	—
	80-56-8	α-pinene	0.0048	0.0052	0.0061	—
Phenols	108-95-2	phenol	—	0.0075	—	—

**Table 2:** Standards for VOC emissions from board materials in different countries and regions.

Standard Number	Standard Name	TVOC Limit Value
HJ 571-2010 [11]	Technical requirement for environmental labeling products—wood based panels and finishing products	≤0.50 mg/(m^2^·h)
GB/T 29899-2024 [26]	Determination of the emission of volatile organic compounds from wood-based panels and furnishing products—small chamber method	—
GB/T 35601-2024 [27]	Green product assessment—wood-based panels and wooden flooring	Green Benchmark Product ≤ 100 μg/m^3^ (7 d) Green Product ≤200 μg/m^3^ (7 d)
GB/T 44690-2024 [28]	Classification of volatile organic compounds emission from wood-based panels and their products	Class I ≤ 200 μg/m^3^ Class II ≤ 400 μg/m^3^
ISO 16000-9:2024 [21]	Indoor air—Part 9: Determination of the emission of volatile organic compounds from building products and furnishing—emission test chamber method	—
ISO 16000-6:2021 [22]	Part 6: Determination of organic compounds (VVOC, VOC, SVOC) in indoor and test chamber air by active sampling on sorbent tubes, thermal desorption and gas chromatography using MS or MS FID	—
EN 16516:2018 [23]	Construction products: Assessment of release of dangerous substances. Determination of emissions into indoor air	—
ANSI/BIFMA M7.1-2011(R2016) [24]	Standard for formaldehyde and TVOC emissions of low-emitting office furniture systems and seating	—
JIS A1901:2015 [25]	Japanese Industrial Standard A1901: Testing methods for determination of the emission of volatile organic compounds and aldehydes for building products—Small chamber method	≤0.4 mg/(m^2^·h) (Small chamber method)

**Table 3:** Changes in the release of various VOCs of particleboard.

Material	Types of VOCs					
	Aldehydes	Alcohols	Ketones	Esters	Benzene Series Compounds	Pinene
PVC-decorated particleboard	−100%	−25%	−93%	2167%	1550%	−43%
PP-decorated particleboard	−99%	−84%	−91%	−100%	0%	−34%
RIP-decorated particleboard	−98%	−100%	−94%	−100%	−34%	−100%

**Table 4:** OAV of VOCs in particleboards before and after decoration.

Substance	Compound	Odor Threshold mg/m^3^	Odor Type	OAV of Particleboards			
				Undecorated	PVC-Decorated	PP-Decorated	RIP-Decorated
Aldehydes	n-pentanal	0.0014	Grassy	1.4286	—	—	—
	hexanal	0.0011	Grassy and apple aroma	24.2727	0.3636	—	4.3174
	heptaldehyde	0.0008	Fruity aroma	0.5000	—	—	—
	nonanal	0.0020	Strong oily and sweet orange aroma	0.5000	—	0.2000	0.2949
	trans-2-octenal	—	—	—	—	—	—
	n-octanal	0.0001	Fruit aroma	22.0000	—	—	—
	decanal	0.0026	Pleasant scent	—	0.1154	—	—
	benzaldehyde	0.0260	Almond odor	—	—	—	0.0226
Alcohols	1-pentanol	0.3600	Odor of fusel oil	0.0011	—	—	—
	1,3-dichloropropanol	—	—	—	—	—	—
	2-ethylhexanol	0.0692	Sweet and light floral fragrance	0.0101	0.0318	0.0087	—
	eucalyptol oil	9.9854	Camphor scent and refreshing herbal taste	0.0004	0.0002	0.0001	—
	linalool	—	Lemon fragrance	—	—	—	—
	α-terpineol	—	—	—	—	—	—
	cedrol	0.5126	Weak wood fragrance with some ointment fragrance	0.0008	—	—	—
	2-propyl-1-heptanol	—	—	—	—	—	—
	1- (2-methoxy-1-methylethoxy) isopropanol	—	—	—	—	—	—
Ketones	acetophenone	0.0026	Strong acacia like sweet fragrance	0.2308	0.1538	0.1923	0.1829
	2-camphanone	—	Camphor wood odor	—	—	—	—
	cyclohexanone	3.5288	Mint	—	0.0143	—	—
	3-hydroxy-2-butanone		Buttery	—	—	—	—
Esters	butyl acetate	0.0758	Intense fruity aroma	—	0.0858	—	—
	2-ethylhexyl acetate	—	—	—	—	—	—
	isooctyl acrylate	—	Ester odor	—	—	—	—
	sec-butyl acetate	0.0114	Joyful fragrance	—	1.0351	—	—
	hydroxyethyl acrylate	—	—	—	—	—	—
	acrylic acid, 6-methylheptyl ester	—	—	—	—	—	—
Benzene Series Compounds	toluene	1.2408	Special fragrant aroma	0.0003	0.0076	—	—
	ethyl benzene	0.7382	Aromatic	—	0.0006	—	—
	xylene	0.2517	Stimulating aromatic scent	—	0.0020	0.0024	0.0018
	4-isopropyl toluene	0.3129	—	0.0006	0.0010	—	—
Pinene	longifolene	0.0024	Wood fragrance and iris like aroma	1.8333	—	—	—
	α-pinene	0.0300	Pine resin aroma	0.1600	0.1733	0.2033	—
Phenols	phenol	0.0216	Sweet taste	—	0.3472	—	—

**Table 5:** Risk values of odorants in particleboards before and after decoration.

Substance	Compound	Risk Values of Odorants in Particleboards			
		Undecorated	PVC-Decorated	PP-Decorated	RIP-Decorated
Aldehydes	n-pentanal	0.0025	—	—	—
	hexanal	0.0297	0.0004	—	0.0052
	heptaldehyde	0.0004	—	—	—
	nonanal	0.0011	—	0.0004	0.0007
	trans-2-octenal		—	—	—
	n-octanal	0.0012	—	—	—
	decanal	—	0.0003	—	—
	benzaldehyde	—		—	—
Alcohols	1-pentanol	0.0005		—	—
	1,3-dichloropropanol	—	—	—	—
	2-ethylhexanol	0.0023	0.0073	0.0020	
	eucalyptol oil	—	—	—	—
	linalool	—	—	—	—
	α-terpineol	—	—	—	—
	cedrol	—	—	—	—
	2-propyl-1-heptanol	—	—	—	—
	1- (2-methoxy-1-methylethoxy) isopropanol	—	—	—	—
Ketones	acetophenone	0.0012	0.0008	0.0010	0.0010
	2-camphanone	—	—	—	—
	cyclohexanone	—	0.1227		
	3-hydroxy-2-butanone	—	—	—	—
Esters	butyl acetate	—	—	—	—
	2-ethylhexyl acetate	—	—	—	—
	isooctyl acrylate	—	—	—	—
	sec-butyl acetate	—	—	—	—
	hydroxyethyl acrylate	—	—	—	—
	acrylic acid, 6-methylheptyl ester	—	—	—	—
Benzene Series Compounds	toluene	0.0001	0.0032	—	—
	ethyl benzene	—	0.0005	—	—
	xylene	—	0.0010	0.0012	0.0008
	4-isopropyl toluene	—	—	—	—
Pinene	longifolene	—	—	—	—
	α-pinene	0.0019	0.0021	0.0024	—
Phenols	phenol	—	0.7500	—	—

## Data Availability

The original contributions presented in this study are included in the article. Further inquiries can be directed to the corresponding author.
